# Effect of Hydraulic Activity on Crystallization of Precipitated Calcium Carbonate (PCC) for Eco-Friendly Paper

**DOI:** 10.3390/ijms10114954

**Published:** 2009-11-11

**Authors:** Jung-Ah Kim, Gi-Chun Han, Mihee Lim, Kwang-Suk You, Miyoung Ryu, Ji-Whan Ahn, Toyohisa Fujita, Hwan Kim

**Affiliations:** 1 Department of Systems Innovation, Graduate School of Engineering, University of Tokyo, 7-3-1 Hongo Bunkyo–ku 113-8656 Tokyo, Japan; E-Mails: tt087279@mail.ecc.u-tokyo.ac.jp (J.-A.K.); tfujita@sys.t.u-tokyo.ac.jp (T.F.); 2 Korea Institute of Geoscience and Mineral Resources (KIGAM), 92 Gwahang-no, Yuseong-gu, Daejeon 305-350, Korea; E-Mails: hsue@dreamwiz.com (G.-C.H.); limmh@paran.com (M.L.); youks@kigam.re.kr (K.-S.Y.); miyung1@snu.ac.kr (M.R.); 3 Department of Material Science and Engineering, College of Engineering, Seoul National University, 599 Gwanak-ro, Gwanak-gu, Seoul 151-742, Korea; E-Mail: hwan94@snu.ac.kr (H.K.)

**Keywords:** hydraulic activity, precipitated calcium carbonate (PCC), crystallization, calcite, aragonite, limestone, eco-friendly paper

## Abstract

Wt% of aragonite, a CaCO_3_ polymorph, increased with higher hydraulic activity (°C) of limestone in precipitated calcium carbonate (PCC) from the lime-soda process (Ca(OH)_2_-NaOH-Na_2_CO_3_). Only calcite, the most stable polymorph, was crystallized at hydraulic activity under 10 °C, whereas aragonite also started to crystallize over 10 °C. The crystallization of PCC is more dependent on the hydraulic activity of limestone than CaO content, a factor commonly used to classify limestone ores according to quality. The results could be effectively applied to the determination of polymorphs in synthetic PCC for eco-friendly paper manufacture.

## 1. Introduction

Limestone resources have been introduced to various fields along with industrial developments. Especially, precipitated calcium carbonate (PCC) manufactured from limestone ore is widely used as a raw material in the paper, paint, rubber, and plastic industries [1]. Among those applications, PCC was introduced as a mild base filler in the acid-free paper (alkaline paper) making process in the 1950s, and in particular, PCC addition to paper is currently necessary for library books [2].

In the traditional paper making process based on wood pulp without adding PCC, paper reacted with chemicals such as oxygen, carbon dioxide, and salts in the air, and was finally acidified by natural acid formation. In addition, the color of acidified paper became yellowish under sunlight, and heat and humidity resulted in the rapid breakdown of the molecules holding the fibers together [3,4]. Biological attacks by a variety of species including algae, bacteria, and fungi also shortened the life expectancy of paper. To address all these problems paper has started to be treated with PCC to neutralize the acids formed by the reaction between wood pulp and chemicals [5].

The biggest benefit of alkaline paper made by adding PCC is a much longer life expectancy than that of traditional paper: more than 1,000 years for the best paper and 500 years for average grades [6]. This is a critical reason that PCC should be used in paper for library materials. Besides that, alkaline paper has several other advantages. It reduces overall manufacture costs and needs fewer corrosive chemicals, thus extending machinery lifetime. Most of all, the process is more environmentally friendly due to following reasons: easier recycling of alkaline paper, less wastewater and by-product production from the process, and less energy consumption due to shorter refining and drying times [6]. Accordingly, the alkaline paper making process using PCC is called an “eco-friendly paper process”.

The demand of high quality PCC in Korea is gradually increasing along with the introduction of the eco-friendly paper process in the paper industry. However, PCC is mostly being imported due to the absence of domestic commercial PCC manufacture technology to meet the demand. It is, therefore, necessary to develop manufacturing technology for high quality PCC in Korea to foment a higher value added industry through the effective utilization of limestone.

In general, PCC manufacture processes are divided into the three following steps: (1) quicklime (calcium oxide, CaO) is obtained by the calcination of limestone ore (calcium carbonate, CaCO_3_), (2) CaO is turned into slaked lime (calcium hydroxide, Ca(OH)_2_) through its reaction with water, which is called “hydraulic process”, and (3) PCC is manufactured by the reaction between Ca(OH)_2_ and carbonate ions (CO_3_^2−^) [1]. Limestone ore mainly consists of CaCO_3_ and also contains minerals such as MgO, Fe_2_O_3_, Al_2_O_3_, and SiO_2_, which are considered as impurities in high quality PCC manufacture [7,8]. Limestone has many classification factors including formation age, CaO content, fineness, crystal particle size, and regional characteristics, etc. [9] and in particular, among these CaO content is the most common factor used in limestone classification.

As mentioned, hydration is one of main processes in PCC synthesis. It is an exothermic reaction as shown in the following chemical reaction [1]:

$$\text{CaO}\;+\;{\text{H}}_{2}\text{O}\;\to \;\text{Ca}{\left(\text{OH}\right)}_{2}\;+\;15.59\;\text{kcal}/\text{mol}$$

The generated heat from the reaction is called “hydraulic activity” and it can be considered as a measure of the degree of hydration of the CaO.

As limestone has been widely used in the cement industry for a long time, various studies on its calcination mechanism have been carried out [8,10,11]. For instance, Ar and Dogu [8] investigated the effects of several conditions such as temperature, residence time, sample size, impurities and amount of vapor on the calcination of limestone. Khraisha and Dugwell [11] studied the calcination of limestone and cement raw meal in a suspension reactor simulating dynamic and thermal conditions within a commercial precalciner [8]. The hydraulic activity of CaO produced from the calcination of limestone has also been widely investigated in the cement industry field [12,13].

Considering that measuring the hydraulic activity of CaO may provide important data for the crystallization of PCC as well as the properties of Ca(OH)_2_ in the PCC manufacture industry, in this study we have focused on the investigation of the relationship between the hydraulic activity of CaO and the crystallization of PCC, and also on the possibility of applying hydraulic activity as a new factor for classification of limestone for eco-friendly paper making, different from the view of hydraulic activity of CaO used in the cement industry.

### 2. Experimental

Nine species (A~I) of limestone ore from Danyang and Yeongwol, Korea, were collected for this study. Chemical components of each limestone ore were analyzed using X-ray fluorescence (XRF; MXF 2000). About 5 kg of each limestone were firstly crushed using a jaw crusher, and were then sieved into the particle sizes of 20 to 35 mm. CaO (120 g) was made by calcination of the sieved limestone (about 200 g) in an electric furnace at 1,000 °C for 2 hours. The calcined CaO was then cooled in a sealed desiccator.

**Figure 1.**
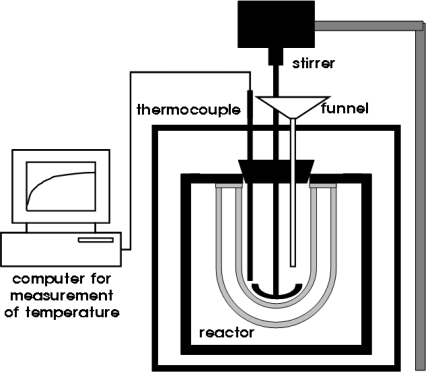
Schematic of a hydration reactor.

CaO was reacted with water to synthesize Ca(OH)_2_ through the hydraulic process. Dried CaO (30 g) was put into a reactor for hydration (see Figure 1). The temperature of the reactor was then fixed at 25 °C in a water bath (Jeio Tech, WB02). Once the temperature of the reactor containing the CaO reached a steady state in the water bath, distilled water (120 mL) at 25 °C was added to the reactor, stirring at 400 rpm for 30 min. After the reaction, each solution was filtered and the filtered precipitate was washed with ethanol three or four times to remove water on its surface. Each precipitate was dried at over 80 °C in an oven and then stored in a vacuum bag. In each hydraulic process, the hydraulic activity was measured by KS E 3077 (Korean Standard Test) [14] to understand its effect on the distribution ratio of the polymorphs (either aragonite or calcite) in synthetic PCC. KS E 3077 describes a standard method for testing hydraulic activity of limestone used to PCC synthesis, and a value of hydraulic activity can be calculated by the following equation:

$$A\;=\;T_{30s}-T_{0}\;=\;\Delta T$$

where A: Hydraulic activity (°C) of limestone

*T_30s_*: Temperature (°C) at 30 seconds from the start of hydration

*T_0_*: Temperature (°C) before the start of hydration

*ΔT*: Value (°C) of temperature change from the start of hydration to 30 seconds

For aragonite synthesis, dry Ca(OH)_2_ produced by hydration on each condition was used and the synthesis method using Ca(OH)_2_-NaOH-Na_2_CO_3_ solution was introduced. This method was suggested by Kim *et al*. [15] as an optimal method to synthesize single phase aragonite. To adjust the CO_3_^2−^ concentration, 150 mL of 0.5 M Na_2_CO_3_ was added at a rate of 3 mL/min to a solution with 50 mL each of 1.5 M Ca(OH)_2_ and 5.0 M NaOH. The reaction temperature and stirring speed were 25 °C and 400 rpm, respectively. The synthesized material under each set of conditions was filtered, washed with ethanol three or four times to remove residual ion components, and then dried in an oven at 80 °C. The dried precipitates were characterized by X-ray diffraction (XRD; MacScience Co. M18XHF-SRA) using CuKα_1_ radiation. Distribution ratios (wt%) of main components in the synthetic PCC were calculated from full width at half maximum of peak on XRD.

## 3. Results and Discussion

**Figure 2.**
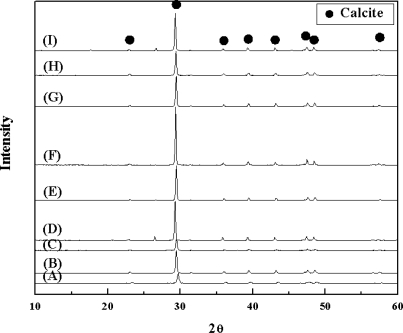
X-ray diffraction (XRD) patterns of the limestone ore samples (A~I): Main peak of each limestone sample presents calcite [• calcite ].

Chemical components of the nine limestone ore samples analyzed using XRF are shown in Table 1. A common factor in limestone classification is CaO content. According to CaO content, limestone ores are classified into the following three groups: high quality (>53 wt%), middle quality (50~53 wt%), and low quality (<50 wt%). All of the limestone ore samples belonged to high quality class, except for the samples G and E, which belong to middle and low quality group, respectively. In the XRD analysis result shown in Figure 2, the main peaks indicate the presence of calcite in the limestone ore samples.

During the hydraulic process after CaO calcination, hydraulic activity was measured by KS E 3077. Overall, higher CaO content tends to show higher hydraulic activity, but some cases were not in accord with this tendency. Specifically, in the case of samples C, D, and F, they are high quality limestone ores with over 55 wt% CaO, but they showed lower hydraulic activities than those of other high quality limestone ore samples. This result indicates that CaO content is a general factor for classifying limestone quality, but it does not determine the hydraulic activity of the limestone.

**Figure 3.**
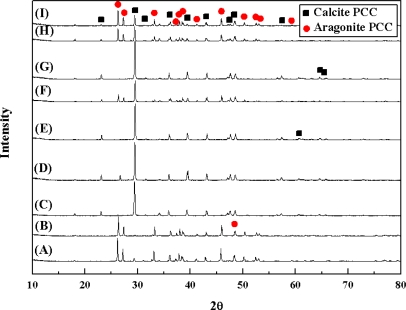
X-ray diffraction (XRD) patterns of final products synthesized from the limestone ore samples A~I [▪ calcite and • aragonite].

It is well known that aragonite synthesis is very difficult because during its synthesis it can rapidly change in solution into calcite, a more stable phase [16]. However, aragonite could be synthesized successfully in this study by applying the optimal synthesis method of [15]. XRD patterns of the synthetic materials are shown in Figure 3, where the main peaks mostly indicate the presence of calcite and aragonite. Especially, higher aragonite peaks are presented in the analysis of final synthetic materials from samples A, B, and I.

Percentages (wt%) of aragonite, calcite, and impurities in each sample were calculated from the XRD patterns in Figure 3 and the values are shown in Table 2. The distribution of PCC reached 90~100 wt% (the sum of aragonite and calcite) in all the cases using the samples A~I, and other impurities were then only 0~9 wt%. Specifically, final synthetic materials prepared from the samples A and B contained more than 90 wt% aragonite, whereas those from the samples C, D, E, and G did not contain any aragonite and most were then calcite. As a result, none of the limestone samples produced single phase aragonite, and most final products consisted of both aragonite and calcite containing some impurities, or single phase calcite (Sample E).

Based on the results of Tables 1 and 2, the crystallization of PCC (aragonite and calcite) synthesized from the samples A~I can be presented as depending on CaO content (wt%). Overall, higher CaO content of a limestone ore sample tends to produce more aragonite, but some specific cases are not consistent with the tendency, like the relationship between CaO content and hydraulic activity. For instance, the samples C, D, and F contained more than 55 wt% CaO, and were thus considered to be high quality limestone ores, but the percentages of aragonite polymorph synthesized from the samples were very low (0~41 wt%), compared to the aragonite produced from other high quality limestone samples (Sample A: 91 wt% and Sample B: 95 wt%). This result therefore implies that the crystallization of PCC does not depend on chemical components of limestone ore, especially CaO content.

**Figure 4.**
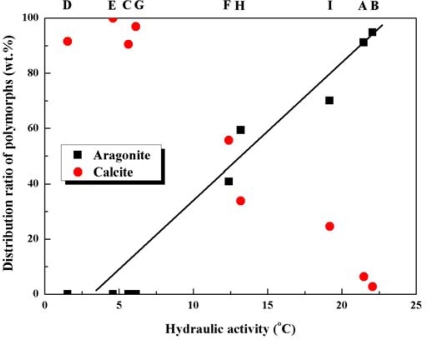
Distribution ratio (wt%) of polymorphs (aragonite and calcite) depending on hydraulic activity (°C) of limestone samples A~I: more aragonite is crystallized with higher hydraulic activity and more calcite with lower hydraulic activity [▪ aragonite and • calcite].

From the above investigations, we found that there was no correlation between CaO content of the limestone ores and both hydraulic activity and the distribution ratio of polymorphs (aragonite and calcite). Accordingly, we also investigated the correlation between hydraulic activity and the crystallization of PCC, and a meaningful result shown in Figure 4 was obtained, where it can be clearly seen that the percentage of aragonite gradually increases with higher hydraulic activity.

This result can be related to the fact that CaCO_3_ polymorphs are determined by the supersaturation rate which is affected by temperature: aragonite is mainly produced at low supersaturation and calcite at high supersaturation [15]. Supersaturation rate is affected by specific surface area (particle size) of Ca(OH)_2_ which depends on the temperature during the hydraulic process. In other words, crystal growth rate of Ca(OH)_2_ becomes fast at high temperature, so that Ca(OH)_2_ has small specific surface area (large particle size). In the PCC synthesis process, the small specific surface area of Ca(OH)_2_ induces a slow ionization rate of Ca(OH)_2_ into Ca^2+^ in solution, and also causes low supersaturation of the solution. As aforementioned, the temperature change generated in the hydraulic process can be considered as hydraulic activity, and the distribution ratio of aragonite and calcite are greatly affected by hydraulic activity as shown in Figure 4. It is hence concluded that the distribution ratio of aragonite and calcite is more related to hydraulic activity than chemical composition (CaO content, wt%) of limestones.

## 4. Conclusions

It was of interest to determine the distribution ratio of CaCO_3_ polymorphs (aragonite and calcite) in synthetic PCC depending on hydraulic activity. No aragonite was crystallized from limestone ore samples with hydraulic activity of less than 10 °C. Aragonite constituted 40~70 wt% of the CaCO_3_ polymorphs synthesized from limestone samples with hydraulic activity in the range of 10~20 °C. In particular, it accounted for more than 90 wt% of the polymorphs from limestone samples with hydraulic activity in more than 20 °C. However, single phase aragonite was not crystallized from any limestone ore samples. An analysis on the chemical composition (*e.g.*, CaO content) of limestone ore has been basically introduced to classify its quality, whereas it is suggested that hydraulic activity should be also considered to determine the distribution ratio of polymorphs (aragonite and calcite) in PCC synthesis. We expect that the results obtained from the present study could be effectively applied to the eco-friendly paper making industry using PCC.

## Figures and Tables

**Table 1.** Chemical components (wt%) of the limestone ore samples (A~I) analyzed using X-ray fluorescence (XRF).

**Limestone Sample**	**Al_2_O_3_**	**CaO**	**Fe_2_O_3_**	**K_2_O**	**MgO**	**MnO**	**Na_2_O**	**P_2_O_5_**	**SiO_2_**	**TiO_2_**	**L.O.I.**
A	0.31	**55.22**	0.16	0.05	0.80	0.01	<0.01	<0.01	0.56	0.01	43.44
B	0.02	**55.87**	0.02	<0.01	0.50	0.01	<0.01	<0.01	0.04	<0.01	43.75
C	1.58	**55.02**	<0.01	<0.01	0.76	<0.01	0.01	<0.01	1.26	0.03	41.31
D	0.27	**55.63**	0.15	0.03	0.16	<0.01	<0.01	0.01	0.33	0.02	43.55
E	1.07	**49.47**	0.34	0.46	1.45	<0.01	0.01	0.02	6.27	0.05	40.93
F	0.24	**55.88**	0.04	0.02	0.15	<0.01	<0.01	<0.01	0.13	0.01	43.58
G	0.27	**52.19**	0.2	0.03	0.37	0.02	0.01	0.01	4.17	0.02	42.34
H	0.04	**53.76**	0.04	0.02	1.27	0.01	<0.01	0.01	0.12	<0.01	44.51
I	0.09	**54.75**	0.04	0.03	0.38	0.01	<0.01	0.01	0.2	0.01	43.73

LOI: loss on ignition.

**Table 2.** Quantitative XRD analysis (wt%) of final products synthesized from the limestone ore samples A~I.

**Limestone ore**	**Components (wt%)**		
	**Aragonite**	**Calcite**	**Others (impurity)**
A	91.1	6.3	2.6
B	94.8	2.7	2.5
C	0	90.5	9.5
D	0	91.5	8.5
E	0	100	0
F	40.8	55.6	3.6
G	0	96.9	3.1
H	59.3	33.8	6.8
I	70.0	24.6	5.4
